# Intracellular and Extracellular Effects of S100B in the Cardiovascular Response to Disease

**DOI:** 10.1155/2010/206073

**Published:** 2010-07-07

**Authors:** James N. Tsoporis, Forough Mohammadzadeh, Thomas G. Parker

**Affiliations:** Division of Cardiology, Department of Medicine, Keenan Research Centre, Li Ka Shing Knowledge Institute, St. Michael's Hospital, University of Toronto, Toronto, ON, Canada M5B 1W8

## Abstract

S100B, a calcium-binding protein of the EF-hand type, exerts both intracellular and extracellular functions. S100B is induced in the myocardium of human subjects and an experimental rat model following myocardial infarction. Forced expression of S100B in neonatal rat myocyte cultures and high level expression of S100B in transgenic mice hearts inhibit cardiac hypertrophy and the associated phenotype but augments myocyte apoptosis following myocardial infarction. By contrast, knocking out S100B, augments hypertrophy, decreases apoptosis and preserves cardiac function following myocardial infarction. Expression of S100B in aortic smooth muscle cells inhibits cell proliferation and the vascular response to adrenergic stimulation. S100B induces apoptosis by an extracellular mechanism via interaction with the receptor for advanced glycation end products and activating ERK1/2 and p53 signaling. The intracellular and extracellular roles of S100B are attractive therapeutic targets for the treatment of both cardiac and vascular diseases.

## 1. The Family of S100 Proteins

S100 proteins entail a multigenic family of calcium binding proteins of the EF-hand type (helix E-loop-helix F). These proteins are called S100 because of their solubility in a 100% -saturated solution with ammonium sulphate at neutral pH. They are small acidic proteins, 10–12 KDa, and contain two distinct EF-hands, 4 *α*-helical segments, a central hinge region of variable length, and the N- and C-terminal variable domains. To date, 25 members of this family have been identified [1]. Of these, 21 family members (S100A1-S100A18, trichohyalin, filagrin, and repetin) have genes clustered on a 1.6-Mbp segment of human chromosome 1 (1q21) while other members are found at chromosome loci 4q16 (S100P), 5q14 (S100Z), 21q22 (S100B), and Xp22 (S100G) [2]. S100 proteins are widely expressed in a variety of cell types and tissues. For example, S100A1 and S100A2 are found in the cytoplasm and nucleus, respectively, of smooth-muscle cells of skeletal muscle [3], S100P is located in the cytoplasm of placental tissue [4, 5], and S100B in cytoplasm of astrocytes of nervous system [6]. However, their expression might be repressed in other cell types by negative regulatory factors which are controlled by environmental conditions. For instance induction of S100B in rat myocardium postinfarction [7] implies that transcription regulation of these proteins is strongly controlled by negative and positive elements [8].

 S100 proteins do not exhibit intrinsic catalytic activity. However, they are calcium sensor proteins and through interaction with several intracellular effector proteins they contribute to the regulation of a broad range of functions such as contraction, motility, cell growth and differentiation, cell cycle progression, organization of membrane-associated cytoskeleton elements, cell survival, apoptosis, protein phosphorylation, and secretion [1, 3, 9]. In order to modulate these types of activities, S100 proteins undergo conformational changes [10]. Upon calcium binding, the helices of S100 proteins rearrange, revealing a hydrophobic cleft, which forms the target protein binding site [11]. Although target binding of S100 proteins is calcium-dependent, calcium independent interactions have been reported [12]. Enzymes are the most common calcium independent target binding for the S100 proteins. For instance, S100B and S100A1 bind with glycogen phosphorylase [13]. The most significant calcium-independent interactions of S100 proteins are their ability to bind to each other. Typically, they are homodimers, but heterodimerization adds to the complexity of this multiprotein family. Each subunit consists of two helix-loop-helix motifs connected by a central linker or so-called hinge region. The C-terminal canonical EF-hand motif is composed of 12 amino acids, whereas the N-terminal S100-specific EF-hand comprises 14 residues [3, 14]. 

 Growing evidence indicates that in addition to intracellular activities, some S100 proteins (e.g., S100B, S100A1, S100A4, S100A8, and S100A9) exhibit extracellular functions [15]. However, secretion has been shown only for S100B, S100A8, and S100A9 [15]. The S100A8/A9 heterodimer is secreted by a novel secretion pathway that depends on an intact microtubule network and acts as a chemotactic molecule in inflammation [16, 17]. The extracellular effects of some S100 proteins require binding to the receptor for advanced glycosylation end products (RAGE) [18–21]. RAGE is a member of the immunoglobulin family of cell surface molecules recognizing multiple ligands including AGE, amphoterin, amyloid-*β*-peptide and *β*-fibrils, S100A12, S100A6, and S100B [22]. The 45-kDa receptor protein consists of 403 amino acids with an extracellular domain (1 variable and 2 constant Ig domains with disulfide bridges), a single transmembrane region, and a short cytosolic tail that triggers signal transduction [23]. RAGE ligands show selective binding to RAGE. S100B tetramer induces receptor dimerization by binding to RAGE [24]. S100B binds to domains V and CI whereas the RAGE ligand S100A6 binds to domains CI and CII [23].

## 2. Noncardiovascular Actions of S100B

S100B is predominantly expressed in astrocytes, oligodendrocytes, and schwann cells. S100B has intracellular and extracellular effects [1]. Intracellularly, S100B regulates the cytoskeletal dynamics through disassembly of tubulin filaments, type III intermediate filaments [1], and binding to fibrillary proteins such as CapZ [25] or inhibiting GFAP phosphorylation when stimulated by cAMP or calcium/calmodulin [26]. S100B interacts in a calcium-dependent manner with the cytoplasmic domain of myelin-associated glycoprotein and inhibits its phosphorylation by protein kinase [27]. It is implicated in the phosphorylation of tau protein [28], inhibition of Ndr kinase activity [29], inhibition of p53 phosphorylation [30], and regulation of the activity of the GTPase Rac1 and Cdc effector, IQGAP [31]. S100B can also be secreted by a number of cell types (e.g., astrocytes, glial cells) [32]. Astrocytes and glial cells secrete S100B, by a complex system involving alterations in intracellular calcium concentration [32]. S100B after secretion, or simply leakage from damaged cells, could accumulate in the extracellular space and/or enter the blood stream and cerebrospinal fluid [33, 34]. The action of S100B is strongly dependent on its extracellular concentration. At nanomolar quantities, it has trophic effects on neurite outgrowth; however, at micromolar concentrations it promotes apoptosis [35, 36]. Such high extracellular levels are detected after brain injury or in neurodegenerative disorders like Down's Syndrome, Alzheimer disease, or encephalitis [37, 38]. Both trophic and toxic effects of extracellular S100B are mediated in the brain by RAGE [36]. In addition to playing a major role in brain physiology [1], S100B has been implicated in cardiovascular development [39] and is considered a biochemical marker for brain injuries after bypass graft surgery [40] and dilated cardiomyopathy [41].

## 3. Cardiovascular Actions of S100B

### 3.1. Intracellular S100B and Myocyte Hypertrophic Gene Expression

**Figure 1:**
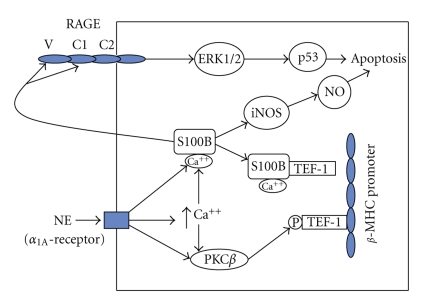
Schematic representation of proposed intracellular and extracellular effects of S100B in cardiac myocytes. Norepinephrine (NE) activation of the calcium-dependent protein kinase C (PKC)-*β*, mediated by the *α*_1_-adrenergic receptor, phosphorylates (P) transcriptional enhancer factor (1) TEF-1, resulting in DNA binding and transactivation of the *β*-myosin heavy chain promoter. By contrast, S100B induction by NE and other hypertrophic signals (not shown) results in calcium-dependent block of PKC-*β* phosphorylation of TEF-1 and inhibition of *β*-MHC transcription. S100B can also induce apoptosis intracellularly via a inducible nitric oxide synthase (iNOS)-NO pathway or it can be secreted and via activation of the receptor for advanced glycation end products (RAGE) (extracellular components V and CI), and induce apoptosis via MEK-ERK1/2-p53 signaling.

The adult cardiac myocyte is terminally differentiated and has lost the ability to proliferate. The myocardium therefore adapts to increasing workloads through hypertrophy of individual cells in response to hormonal, paracrine, and mechanical signals [42, 43]. This process is initially compensatory but it can progress to irreversible enlargement and dilatation of the ventricle resulting in heart failure [44]. Myocyte hypertrophy is accompanied by the down-regulation of adult *α*-myosin heavy chain and a program of fetal gene reexpression including the embryonic *β*-myosin heavy chain (MHC), the skeletal isoform of *α* actin (skACT), and atrial natriuretic factor (ANF) [45, 46]. This response can be reproduced *in vitro* in cultured neonatal cardiac myocytes by treatment with a number of trophic factors including peptide growth factors and *α*_1_-adrenergic agonists [7]. Negative modulators of the hypertrophic response are essential to maintain a balance between compensatory hypertrophy and unchecked progression. Experimental evidence suggests that S100B acts as an intrinsic negative regulator of the myocardial hypertrophic response [47–49]. S100B not normally expressed in the myocardium, is induced in the peri-infarct region of the human heart after myocardial infarction [47] and in rat heart commencing at day 7 following myocardial infarction as a result of experimental coronary artery ligation [7]. In cultured neonatal rat cardiac myocytes, transfection of an expression vector encoding the human S100B protein inhibits the *α*_1_-adrenergic induction of the fetal genes *β*- MHC and the skACT [7]. The inhibition of *α*_1_-adrenergic induction is selective as S100B does not inhibit the capacity of thyroid hormone to induce *α*-myosin heavy chain. To establish that S100B blocked *α*_1_-adrenergic induction of *β* -MHC and skACT by interrupting the PKC signaling pathway, the interaction between forced S100B expression and a constitutively activated mutant of PKC*β* referred to as *δ*PKC*β*  was tested [50]. *δ*PKC*β* transactivated the *β*-MHC and skACT genes supporting the notion that the *α*_1_-adrenergic induction of these genes is mediated by activation of the class-I PKC isoform *β*-PKC [7, 50]. Forced S100B expression could only block *δ*PKC*β*-induced transaction of *β*-MHC and skACT amidst concomitant treatment with an *α*_1_-adrenergic agonist or augmented extracellular calcium suggesting that the capacity of S100B to modulate the hypertrophic phenotype is calcium dependent [7]. The transcription factors TEF-1 (transcription factor-1) and related TEF-1 (RTEF-1) upon phosphorylation by PKC*β* bind to MCAT elements on the skACT and *β*-MHC promoters and activate transcription [51]. In cotransfection experiments, forced expression of S100B inhibited the activation of the skACT and *β*-MHC promoters by overexpression of TEF-1 (unpublished observations). A direct interaction between S100B and TEF-1 was demonstrated using a coimmunoprecipitation strategy (unpublished observations). These data suggest that S100B modulates the activation of the fetal genes by direct binding to TEF-1. In addition to TEF-1, S100B interacts in a calcium-dependent manner with the giant phosphoprotein AHNAK/desmoyokin in cardiomyocytes and smooth muscle cells [49]. In cardiomyocytes, AHNAK plays a role in cardiac calcium signaling by modulating L-type calcium channels in response to *β*-adrenergic signaling [52, 53]. The S100B/AHNAK interaction may participate in the S100B-mediated regulation of cellular calcium homeostasis [53]. Whether there is any relationship between S100B-mediated effects on calcium fluxes and S100B-dependent inhibition of the *α*_1_-induction of the hypertrophic phenotype remains to be elucidated. The function of the S100B/AHNAK interaction in smooth muscle cells is currently unknown. In the myocardium, S100B expression is transcriptionally controlled dependent on positive (−782/−162 and −6,689/−4,463) and negative (4,463/−782) elements upstream of the transcription initiation site, selectively activated by *α*_1A_-adrenergic signaling via PKC*β* and inhibitory and stimulatory DNA binding by transcription factors, TEF-1 and related RTEF-1, respectively [8] (Figure 1). This suggests that the same *α*_1_-adrenergic pathway that initiates and sustains the hypertrophic response in cardiac myocytes by activating PKC signaling and which is subject to negative modulation by S100B also induces the S100B gene.

### 3.2. Intracellular S100B, Cardiovascular Hypertrophy and Apoptosis

 To provide a physiologic model of S100B overexpression effects, transgenic mice were created that contained multiple copies of the human gene under the control of its own promoter. These animals demonstrate normal cardiac structure, and neuronal, but no basal cardiac expression of the transgene. In S100B transgenic mice, after chronic *α*_1_-adrenergic agonist infusion, S100B is detected in the heart and increased in the vasculature [49]. In addition, the myocyte hypertrophy and arterial smooth muscle cell proliferation normally evoked in the heart and vasculature, respectively, in response to *α*_1_-adrenergic stimulation in wild-type mice were abrogated in S100B transgenic mice [49]. In knockout mice, *α*_1_-adrenergic agonist infusion provoked a potentiated myocyte hypertrophic response and augmented arterial smooth muscle cell proliferation. Furthermore, in knockout mice, both the acute and chronic increases in blood pressure in response to *α*_1_-adrenergic agonist infusion were attenuated compared with wild-type mice [49]. To determine whether this inhibition is generalizable to other hypertrophic stimuli, transgenic and knock-out animals were subjected to descending aortic-banding to produce pressure-overload. Aortic banding for 35 days increased left ventricular (LV)/body weight (BW) ratio in CD-1 controls (4.61 ± 0.06 g/kg versus 3.44 ± 0.16 g/kg in sham operated, *P* < .05, *n* = 6) and produced no hypertrophy in S100B transgenic mice (3.37 ± 0.12 g/kg versus 3.26 ± 0.11 g/kg in sham operated, *P* < .05, *n* = 8) and excessive hypertrophy in knock-out mice (5.12 ± 0.24 g/kg versus 3.19 ± 0.13 g/kg in sham operated, *P* < .05, *n* = 6). Similarly, thirty five days after experimental myocardial infarction, the S100B knockout mice mounted an augmented hypertrophic response compared to wild-type mice [48]. Fetal gene expression was induced to a greater magnitude in knockout mice compared to wild-type mice. The S100B transgenic mice did not develop the hypertrophic phenotype but demonstrated increased apoptosis in the peri-infarct region compared to wild-type and knockout mice. The postinfarct hypertrophic response in the myocardium is initiated by multiple trophic signals that include the state of local and systemic sympathetic hyperactivity through *α*_1_-adrenergic stimulation [54]. These studies in S100B transgenic and knockout mice complement the culture data and support the hypothesis that S100B acts both as an intrinsic negative regulator of hypertrophy and an apoptotic agent. Intracellular S100B may modulate the apoptotic responses of postinfarct myocytes by activating a transcriptionally inducible form of nitric oxide synthase (iNOS) and production of nitric oxide (NO) [55] as has been described for astrocytes [35]. Forced expression of S100B may induce iNOS, NO production, and apoptosis. Thus NO could be an intermediate pathway in the induction of apoptosis by intracellular S100B (Figure 1). Similar to S100B, S100A6 is upregulated in post-infarct myocardium and is selectively induced by TNF-*α* and serves to limit myocyte apoptosis [56]. S100B colocalizes with S100A6 in cardiac muscle [57], suggesting that heterodimerization may have distinct phenotypic consequences.

### 3.3. Extracellular S100B and Myocyte Apoptosis

 Increasing evidence suggests that S100B plays a role in the regulation of apoptosis in post-MI myocardium by an extracellular mechanism after cellular release from damaged myocytes and interaction with RAGE [58]. Exogenously administered S100B to neonatal rat cultures induced apoptosis in a dose-dependent manner beginning at 0.05 *μ*mol/L, a local or regional concentration that may be achieved in the peri-infarct myocardium [48]. Similarly, S100B at dose ≥0.05 *μ*mol/L induced neuronal cell death [59]. Myocyte apoptosis is accompanied by cytochrome C release from mytochondria to cytoplasm, increased expression and activity of pro-apoptotic caspase-3, decreased expression of anti-apoptotic Bcl-2, and phosphorylation of ERK1/2 and p53 [58, 60, 61] (Figure 1). Transfection of a full-length cDNA of RAGE or a dominant-negative mutant of RAGE resulted in increased or attenuated S100B-induced myocyte apoptosis, respectively, implicating RAGE dependence. Inhibition of MEK signaling or overexpression of a dominant negative p53 inhibits S100B-induced myocyte apoptosis. This implies that RAGE activation by S100B increases MEK MAPK kinase signaling, p53 phosphorylation at serine 15, and p53-dependent myocyte apoptosis (Figure 1).

 The effects of S100B on myocyte apoptosis stand in contrast to S100A1, the most abundant S100 protein expressed in cardiac muscle under basal conditions [62]. S100A1 exhibits increased expression in compensated hypertrophy, decreased expression in human cardiomyopathy, and downregulation following experimental myocardial infarction [63, 64]. S100A1 knockout mice showed elevated systemic blood pressure, reduced endothelium-dependent vasorelaxation, and decreased survival after myocardial infarction [65, 66]. Like our proposed mechanism for S100B release, S100A1 is released into the extracellular space in the setting of myocardial injury and can bind RAGE [58]. Unlike S100B, extracellular S100A1 inhibits apoptosis independent of RAGE [67] or by RAGE signaling by interacting with a different extracellular domain of RAGE as has been shown with other RAGE ligands [23]. Thus, S100 proteins may differentially regulate myocardial structure and function. Given the capacity of S100A1 and S100B to heterodimerize, phenotypic consequences may depend on the availability and stoichiometry of S100A1 and S100B homodimers and heterodimers.

## 4. Concluding Remarks

 In conclusion, the S100 family constitutes the largest subgroup of the EF-hand family of calcium-binding proteins with 25 members. S100 proteins have been implicated in pleiotropic calcium-dependent cellular events, with specific functions for each of the family members. S100B is induced in peri-infarct myocytes postmyocardial infarction in human subjects and experimental rodent models of myocardial infarction and in response to *α*_1_-adrenergic stimulation. S100B plays an important role in negative intrinsic regulation of aortic smooth muscle cell proliferation, cardiac myocyte hypertrophy, and, via RAGE ligation, apoptosis. The intracellular and extracellular roles of S100B are attractive therapeutic targets for the treatment of both cardiac and vascular diseases.
